# Preliminary study on activity monitoring using an android smart-watch

**DOI:** 10.1049/htl.2014.0091

**Published:** 2015-02-25

**Authors:** Vijayalakshmi Ahanathapillai, James D. Amor, Zoe Goodwin, Christopher J. James

**Affiliations:** 1 International Digital Laboratory, Institute of Digital Healthcare – WMG, University of Warwick, Coventry, CV4 7AL, UK; 2 Warwick Engineering in Biomedicine, School of Engineering, University of Warwick, Coventry, CV4 7AL, UK; 3 Management Science Department, University of Strathclyde, Glasgow, G1 1XQ, UK

**Keywords:** assisted living, health care, mobile computing, patient monitoring, activity monitoring, android smart-watch, independent living project, USEFIL project, unobtrusive smart environments-for-independent living project, assistive technology, wrist wearable unit, WWU, assisted living, PA parameter, activity level

## Abstract

The global trend for increasing life expectancy is resulting in aging populations in a number of countries. This brings to bear a pressure to provide effective care for the older population with increasing constraints on available resources. Providing care for and maintaining the independence of an older person in their own home is one way that this problem can be addressed. The EU Funded Unobtrusive Smart Environments for Independent Living (USEFIL) project is an assistive technology tool being developed to enhance independent living. As part of USEFIL, a wrist wearable unit (WWU) is being developed to monitor the physical activity (PA) of the user and integrate with the USEFIL system. The WWU is a novel application of an existing technology to the assisted living problem domain. It combines existing technologies and new algorithms to extract PA parameters for activity monitoring. The parameters that are extracted include: activity level, step count and worn state. The WWU, the algorithms that have been developed and a preliminary validation are presented. The results show that activity level can be successfully extracted, that worn state can be correctly identified and that step counts in walking data can be estimated within 3% error, using the controlled dataset.

## 1 Introduction

There is a growing worldwide trend for people to live longer, with an increasing population of older people, aged 60 and over [1], which in turn leads to two important observations. First, as the number of older people in the population increases, the number of people with age related health conditions increases. Secondly, as the proportion of older people increases, the proportion of people able to provide care decreases. This impacts the number of available carers and the relative amount of money with which care can be provided [2].

These effects combine to create a pressure on society to provide healthcare for its older population using relatively fewer resources per person. However, in parallel with this pressure, the last decade has seen a rapidly expanding boom in technology. As the price of technology falls and the performance increases, technology is being used to solve a number of emerging challenges. There is a movement towards using technology to provide or augment healthcare [3] and to tackle emerging challenges around the delivery of healthcare.

The Unobtrusive Smart Environments for Independent Living (USEFIL) project [4] is aiming to utilise established and emerging technology to develop a system to assist independent living for older people. The USEFIL project will combine off-the-shelf devices to create an integrated independent living system. Although this project is targeted at the older population, the methods described in this Letter are suitable for all adults. Specifically, this Letter focuses on the wrist wearable unit (WWU) and the useful activity related measures that are obtained in order to monitor the activity of a person. The intention of this work, which builds on the authors' previous work [5, 6], is to present the algorithms used to measure activity level, step count and worn state for a WWU in the USEFIL system, and show preliminary validation in normal controls. It should also be noted that in this Letter it is the identification of a measure of the level of physical activity (PA) that is presented and not an accurate classification of different activities themselves.

## 2 Wrist wearable unit

As part of the USEFIL system a WWU is being developed. The requirements for the WWU in the USEFIL system broadly encompass gathering data on the PA of the user, communicating automatically with the USEFIL system and allowing the use of customised algorithms. An analysis of the market reveals that there are a number of devices available in the health and activity monitoring area and fall into three categories; sensor platforms, health and lifestyle devices and smart-watches. Of these, it is smart-watches that meet the key criteria for USEFIL. Sensor platforms, such as the Actigraph [7] typically do not have the communication or algorithmic flexibility required and health and lifestyle devices, such as the Fitbit [8] are typically locked to proprietary algorithms and data interfaces.

**Figure 1:**
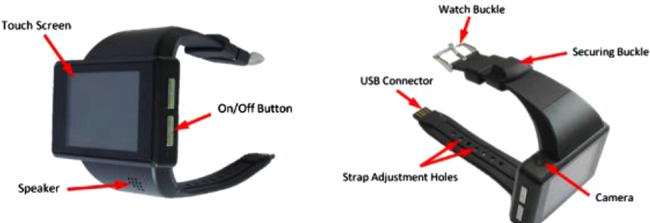
Android Watch-Phone used as WWU in USEFIL project to monitor activity

To meet the system requirements, an off-the-shelf Android-based smart-watch, the Android Z1, shown in Fig. 1 is being used. The Z1 weighs 160 g and the dimensions of the device are 64 mm × 42 mm × 14.5 mm, with a 50.8 mm capacitive touch screen of 320 × 240 pixel resolution. The base chip is a MT6516 which runs a 416 MHz processor with 256 MB RAM and 8 GB internal memory. The Z1 has full Bluetooth, WiFi and GSM connectivities as well as GPS and accelerometer sensors. The accelerometer is tri-axis with a range of ± 2 g. The device is limited by its 800 mAh battery, which lets the device record data from the accelerometer of the device, continuously, for ∼5 h.

### 2.1 Integration with USEFIL

The USEFIL system provides a number of services and applications to assist independent living. One key service is a high level decision support system (DSS) that will combine the sensor inputs of the USEFIL system and provide suggestions that benefit the healthcare of the user. The WWU acts as a key sensor in this part of the system and provides information on the PA of the user.

The WWU pulls data from the accelerometers and runs a suite of algorithms on the data to extract the activity parameters. These parameters are sent back into the USEFIL system through the WWUs WiFi connection and integrated into the USEFIL System DSS.

### 2.2 Activity monitoring

As PA is related to the health of the person, assessing PA levels continuously is essential in order to recognise the change in health status [9]. Also, in order to meet the amount of moderate and vigorous PA for older adults, recommended by the World Health Organization (WHO) [10], a quantitative or direct measure for activity level is preferred in comparison to self-reported measures [11]. Accelerometers are widely used to monitor PA and the general considerations for choosing an accelerometer for use in studies with older adults given by Murphy [12] include the lifestyle PA and step count.

Although, the placement of accelerometers is usually chosen based on the application, for example, whole body movements (chest, sternum, underarm or waist), leg movement (shin, ankle) and Parkinsonian tremor (wrist), for long-term unobtrusive monitoring, as in USEFIL, a simple system using only one sensor attached to the wrist is generally preferred, especially for older people [13]. This was reflected in the responses obtained in early focus groups – potential users had a definite preference towards the wrist mounted devices.

PA monitoring to extract various PA parameters using accelerometers is an active area of research. Previous research on activity levels has shown that, as activity intensity increases, the root-mean square (RMS) of the acceleration signal increases, meaning that the use of RMS score for a measure of activity is valid [14]. In [15], it is shown that the vector magnitude of the tri-axis accelerometers correlates with calorific energy expenditure for a range of activities.

Step counts are widely used in research to relate activity to the health state, promote healthy lifestyle [16] and also to show that PA can affect health outcomes, particularly in chronic conditions [17]. Some step count detection algorithms include Pan and Tompkins [18], originally designed to detect QRS complexes, but more recently applied to step count [19], a peak detection method based on combined dual-axial signals (*x* and *z* axes) [19], threshold-based peak detection [20] and template matching [19].

As mentioned previously, the WWU gathers data from its accelerometers and uses this to perform activity monitoring. In a system such as USEFIL, activity level information combined with step counts can be used to determine activity patterns and daily rhythms. Furthermore, through the detection of PA, it is possible to extrapolate patterns in behaviour, such as sleep-wake cycles, which act as key indicators to health status. For example, a person who is ill might spend longer in bed.

## 3 Data processing

**Figure 2:**
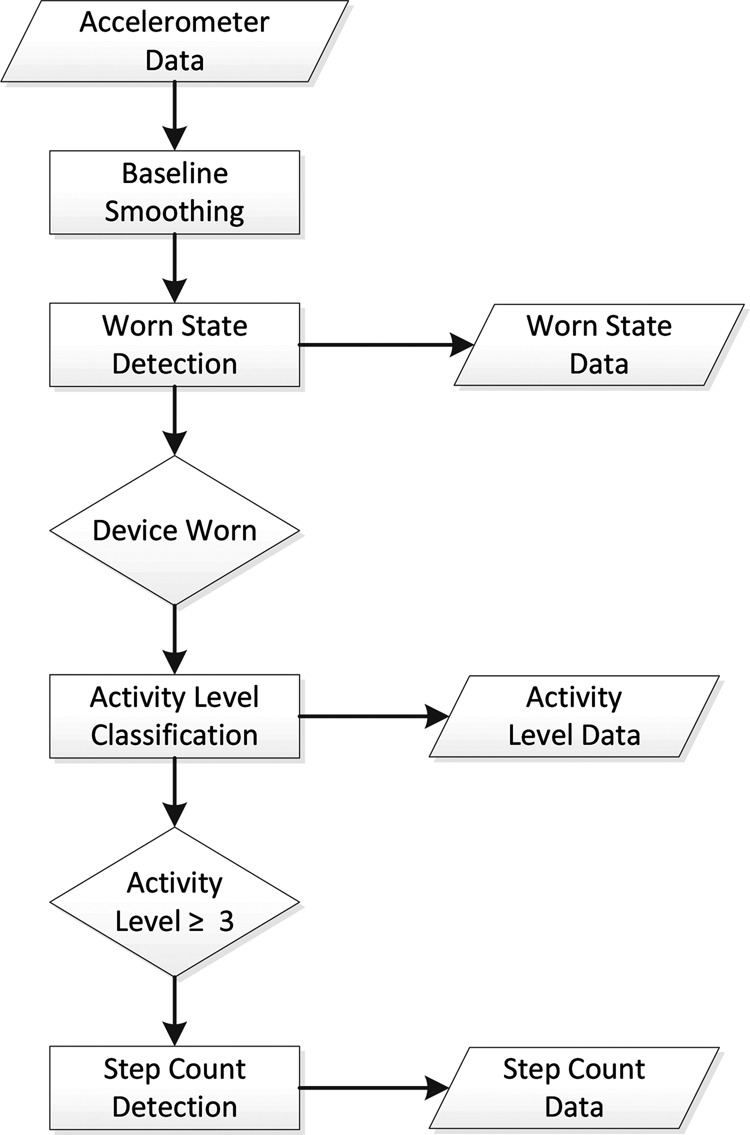
High-level overview of pathway to extract activity parameters from accelerometer data from WWU

To enable the smart-watch to function as the WWU in the USEFIL system a bespoke app has been written for the device to perform on-going data collection and processing. The app can be configured in a number of ways to adjust sampling frequency and sampling periods so that battery life can be managed. The high level overview of the data processing pathway is presented in Fig. 2.

There is an initial smoothing step, followed by a cascade of activity parameter calculations to provide the suite of measurements the WWU produces. It should be noted that if the device is not worn, no further processing is performed, as any measures produced if the WWU was not worn would be invalid. Similarly, if the WWU does not detect any walking events in the data, no step count is derived. The remainder of this Section presents the specific data processing algorithms that are used in each calculation step of the data processing pathway.

### 3.1 Data acquisition

The WWU is configured to record data from the accelerometers at a frequency specified as *f*_s_ for an epoch length of *τ* seconds. Data from the accelerometers in three axis of acceleration are represented as

(1) $${\bi x} = \left\{{x_1 \comma \; x_2 \comma \; \ldots \comma \; x_n } \right\}\eqno\lpar 1\rpar $$

(2) $${\bi y} = \left\{{y_1 \comma \; y_2 \comma \; \ldots \comma \; y_n } \right\}\eqno\lpar 2\rpar $$

(3) $${\bi z} = \left\{{z_1 \comma \; z_2 \comma \; \ldots \comma \; z_n } \right\}\eqno\lpar 3\rpar $$

where ***x***, ***y*** and ***z*** are vectors containing data points for the three axes of motion, *x_i_*, *y_i_* and *z_i_* represent a data point in one of the axes and *n* is the number of samples in the epoch. For any given epoch, *n* can be approximately calculated as

(4) $$n = \left\lfloor {\tau \times f_{\rm s} } \right\rfloor \eqno\lpar 4\rpar $$

The exact number of samples will differ slightly since the Android platform does not maintain a strict sampling rate because sensor reading is not a priority for the platform. The result is that data are approximately sampled to the requested rate, but this rate is slightly variable, especially when the device's processor is under heavy load from other processes. In practice, this means that sampling rates are occasionally slower than requested for a few samples and occasionally faster than requested by a few samples. Data could be interpolated to obtain a uniform sampling rate, but over any reasonably short time frame, the data can be assumed to be uniform with no consequence. The sampling provided is consistent enough for the practical purposes in this Letter.

### 3.2 Baseline smoothing

Baseline smoothing has the specific purpose of removing analogue-to-digital conversion noise from accelerometery signals in a computationally efficient manner. It acts to suppress the microfluctuations in the signal that are caused by the analogue-to-digital conversion in the device and results in truly ‘flat’ signals where there is no movement, whilst preserving areas of genuine movement in the signal. The approach is shown here for the *X*-axis. Initially the baseline, *b*, is set such that

(5) $$b = x_1 \eqno\lpar 5\rpar $$

Subsequently, the following update rules are used to populate the output ***x****′*

(6) $$x^{\prime} _i = \left\{{\matrix{ b & {{\rm if}\, \, \left\vert {x_i - b} \right\vert \lt T_b } \cr {x_i } & {{\rm otherwise}} \cr } } \right.\eqno\lpar 6\rpar $$

(7) $$b = \left\{{\matrix{ b & {{\rm if}\, \, \left\vert {x_i - b} \right\vert \lt T_b } \cr {x_i } & {{\rm otherwise}} \cr } } \right.\eqno\lpar 7\rpar $$

where *T_b_* is the threshold value (typically *T_b_* = 0.5) and *x′_i_* are the values after smoothing. The same operation is repeated on the *Y* and *Z* axes.

### 3.3 Worn-state detection

The worn state (worn or not worn) of the WWU is determined by detecting periods in the accelerometry signal when the device is stationary over time. During these stationary periods, the device is assumed to be not worn. The primary feature of these periods in the accelerometry signal is the absence of any content in the signal aside from the constant reading observed in one or more axis due to the effect of gravity. The algorithm removes the constant component from the RMS of the accelerometry signal and examines the subsequent signal for movement. If none is found, a period of not worn is assigned. The root mean square combination of the *X*, *Y* and *Z* axes after removing the device noise is defined as

(8) $${\bi r} = \left\{{r_1 \comma \; r_2 \comma \; \ldots \comma \; r_n } \right\}\eqno\lpar 8\rpar $$

where

(9) $$r_i = \sqrt {\displaystyle{{x_i^{{\prime}2} + y_i^{{\prime}2} + z_i^{{\prime}2} } \over 3}} \eqno\lpar 9\rpar $$

The gravity component of the signal is removed from ***r*** using a sliding window of 3 s duration. The window has a length *W* samples, where *W* is always odd. An additional parameter, *h*, for just under half the window length is defined as

(10) $$h = \displaystyle{{W - 1} \over 2}\eqno\lpar 10\rpar $$

Using these parameters, the sliding window operation to provide the vector ***r′*** is defined as

(11) $$r^{\prime}_i = \; \left\{{\matrix{ {\matrix{ {r_i - \displaystyle{1 \over W}\; \; \sum\nolimits_{\,j = 1}^W {r_j } \comma \; } & {\; \; \; \; \; \; \; \; 1 \le i \le h\; \; \; \; \; \; \; \; \; \; \; \; } \cr } } \cr {\matrix{ {r_i - \displaystyle{1 \over W}\sum\nolimits_{\,j = i - h}^{i + h} {r_j } \comma \; \; \; } & {\; \; \; \; \; \; h \lt i \le \lpar n - h\rpar } \cr } } \cr {\matrix{ {r_i - \displaystyle{1 \over W}\sum\nolimits_{\,j = n - W + 1}^n {r_j } \comma \; \; } & {\lpar n - h\rpar \lt i \le n} \cr } } \cr } } \right.\eqno\lpar 11\rpar $$

From the vector ***r′***, an index value *v_W_* is calculated as

(12) $$v_W = \displaystyle{1 \over n}\mathop \sum \limits_{i = 1}^n r^{\prime}_i \eqno\lpar 12\rpar $$

and compared with a threshold value *T_w_* (typically *T_w_* ∼ 0.02). If the index value is greater than the threshold then the WWU is determined to be worn. If not, then the WWU is determined to be not worn.

### 3.4 Activity level classification

Activity level is classified through a numerical analysis of the gravity subtracted vector ***r′***. The activity index value *v*_A_ is calculated as

(13) $$v_{\rm A} = \displaystyle{1 \over n}\mathop \sum \limits_{i = 1}^n \left\vert {r^{\prime}_i } \right\vert \eqno\lpar 13\rpar $$

The index value *v*_A_ could be used as is, but for the USEFIL system the value is manipulated to provide a single integer and to expand the lowest region of the activity index value. The mapping from an index value of activity level value is given by

(14) $$A = \left\{{\matrix{ {\left\lceil {4v_{\rm A} } \right\rceil } & {{\rm if}\, \, 0 \lt v_{\rm A} \le 0.5} &\cr {\left\lceil {2v_{\rm A} } \right\rceil + 1} & {{\rm if}\, \, v_{\rm A} \gt 0.5} \cr } } \right.\eqno\lpar 14\rpar $$

The accelerometer recording is considered to be an active period, such as walking or running if the activity levels are greater than or equal to 3. During the active period of the data, the number of steps is detected using the algorithm below.

### 3.5 Step count detection

**Figure 3:**
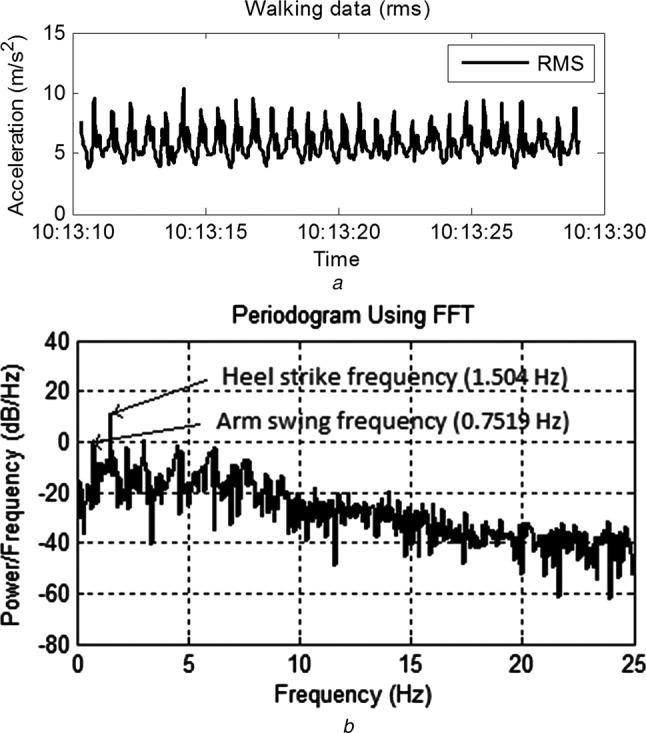
RMS combination of walking data from healthy young adult and periodogram showing frequencies for heel strike and arm swing *a* RMS combination of walking data from healthy young adult *b* Periodogram showing frequencies for heel strike and arm swing

Step count detection is performed on the vector *r* and focuses on extracting the walking frequency from the data. In general, for accelerometery data taken from the wrist, for walking in healthy controls, there can be observed one or two dominant frequencies. These correspond to the heel strike frequency, which is always present, and arm swing frequency, which may not be present. These key frequencies are denoted as *f*_h_ and *f*_a_, and have peak heights *p*_h_ and *p*_a_. Further observations can be made from this data. First

(15) $$\min \lpar p_{\rm h} \comma \; p_{\rm a} \rpar \ge 0.75 \times {\rm max}\lpar p_{\rm h} \comma \; p_{\rm a} \rpar \eqno\lpar 15\rpar $$

which says that the two peaks are similar in height. Secondly

(16) $$\; f_{\rm h} \sim 2f_{\rm a} \eqno\lpar 16\rpar $$

which says that the heel strike frequency is roughly twice that of the arm swing frequency. Furthermore, neither *p*_h_ nor *p*_a_ is reliably greater than the other. This has not been tested on walking pattern from older people. Fig. 3*a* shows the walking data (RMS combination of the three axes). The arm swing and heel strike frequencies for this data can be seen in Fig. 3*b*.

For determining step count, the frequency of interest is *f*_h_. The initial problem therefore is to extract the two dominant frequencies from ***r*** and determine which ones correlate to *f*_h_ and *f*_a_. Initially, ***r*** is transformed via a fast Fourier transform (FFT) into the frequency domain, to give the vector

(17) $${\bi f} = {\rm FFT}\lpar {\bi r}\rpar \eqno\lpar 17\rpar $$

The two dominant peaks are extracted from ***f*** to give two frequency–height pairings, (*f_x_*, *p_x_*) and (*f_y_*, *p_y_*), which are returned in order of peak height such that *p_x_* > *p_y_*.

Following the extraction of the two dominant frequencies, the observations (15) and (16) can be used to determine which peak corresponds to *f*_h_. If (15) does not hold true, substituting *p_x_* and *p_y_* in place of *p*_h_ and *p*_a_, then only one frequency is present, and *f_x_* corresponds to the target frequency *f_h_*. If (15) does hold true then both frequencies may be present and (16) can be used to derive

(18) $$1.5 \times \min \lpar f_x \comma \; f_y \rpar \lt \max \lpar f_x \comma \; f_y \rpar \lt 2.5 \times \min \lpar f_x \comma \; f_y \rpar \eqno\lpar 18\rpar $$

which holds true if one of the two frequencies *f_x_* and *f_y_* is roughly double the other. If (18) is true then the target frequency corresponds to max(*f_x_*, *f_y_*). If (18) does not hold true then it is assumed that *f_y_* is an anomaly and that the target frequency is *f_x_*.

Once the target frequency has been identified, ***f*** is windowed with a 0.4 Hz band pass around *f*_h_. A window of length 2*ω* + 1 is used where *ω* is calculated to create the 0.4 Hz band and used to obtain ***f′*** such that

(19) $$f^{\prime}_i = \left\{{\matrix{ {\,f_i } & {{\rm where}\, \, i = f_{\rm h} \pm \omega } \cr 0 & {{\rm elsewhere}} \cr } } \right.\eqno\lpar 19\rpar $$

An inverse FFT is applied to ***f′*** and followed by smoothing with a window size of 5. This results in a smoothed vector that contains all the information from ***r*** in a 0.4 Hz band around the primary walking frequency, which we denote as r*.

The peaks are detected in r* by identifying all the local maxima; defined as a point, $r_i^\ast $, that satisfies the condition

(20) $$r_{i - 1}^\ast \lt {r_i^\ast }\gt r_{i + 1}^\ast \eqno\lpar 20\rpar $$

A further step is taken to remove any peaks that fall within 0.3 s of a preceding peak. The goal of this being to remove any erroneous peaks caused by the toe strike in the walking pattern that is not filtered out by the band pass filter. The number of steps is then the remaining number of local maxima.

## 4 Validation methodology

This Section presents the methodology that has been used to validate the algorithms used in the WWU. The worn state detection, activity level classification and step count validation methodologies are all presented here.

### 4.1 Worn-state detection

The worn-state detection algorithm was validated with a set of tests examining different wearing modalities. The WWU was set to collect data continuously in 1 min segments and to report both the raw data and the worn state. Four tests were performed with the device being worn, not worn, put on and taken off with three repetitions of each. A single participant was used as the algorithm is principally detecting when the device is not worn. For the worn segments of the test, different intensities of activity were used, although of principal interest is sedentary activity, as this is most likely to produce false positives. The results of the tests were analysed against the known truth for each test.

### 4.2 Activity level classification

The activity level classification algorithm was validated with a longitudinal test using a single participant (healthy male, 29 years old). The WWU was set to collect data continuously and provide the activity level as an output. A ground truth was established at a high level for the duration of the test by asking the participant to log his activity manually. It should be noted that because of the nature of the activity level classification it is difficult to provide an objective measure of the validity of the algorithm. Activity level is a subjective assessment; it should correlate with energy expenditure, but it is not possible to determine that any particular section of activity scores a 4 or a 2, for example.

Several studies have shown that activity intensity (energy expenditure) is correlated to the RMS value of the acceleration signal. Work by Easton *et al.* [15] has been presented that shows that the RMS value correlates with energy expenditure and a study by Amor and James [5] has shown that activities of increasing intensity produce accelerometery data with an increasing signal power (signal power itself is correlated to the RMS of the signal). On the basis of this work, we have chosen to validate the algorithm subjectively, rather than in any objective manner.

### 4.3 Step count

Testing of the step count algorithm was performed on normal controls (*n* = 20, 13 male, 7 female, aged 20–50, height 160–191 cm, weight 47–110 kg). The placement of the WWU was on the non-dominant wrist and data were sampled at 50 Hz. The following four activities were performed by each participant:

1. 100 step walk at normal pace.
2. 100 step walk at 1 Hz pace.
3. Up-stairs walk at normal pace.
4. Down-stairs walk at normal pace.

The performed activities were designed to provide a broad range of walking motion for subsequent analysis and the 1 Hz pace chosen specifically to provide a slower walking motion. However, this had the unintentional effect of altering the participants’ normal gait. Step counts for the stair sections were manually recorded.

The percentage error between ground truth and algorithmically determined step count was calculated and a two one-sided test (TOST) used to check for equivalence – that is, that percentage errors were statistically equivalent to zero. The threshold for zero equivalence was set at 3%, in line with previous studies [21] and a 95% confidence level was used.

The step count algorithm was further tested on the WWU data, sampled at 16 and 5 Hz, to see if the algorithm could detect the steps accurately at reduced sampling rates.

## 5 Results and discussion

### 5.1 Worn and not worn detections

**Figure 4:**
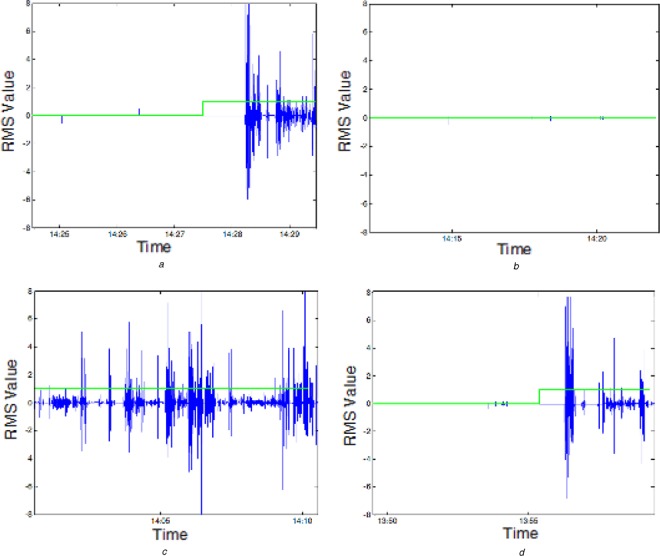
Graphs of activity and worn status indication *a* Worn at end of signal *b* Not worn throughout *c* Worn throughout *d* Worn at end of signal RMS signal shown in blue; worn status shown in green where 1 indicates worn and 0 indicates not worn

The results of the worn-state testing are shown in Fig. 4, which shows the correct identification of worn state in all test cases. There are some periods of non-agreement between ground truth and determined worn state which occur because of the analysis of data in 1 min epochs. If the device is actually worn at any time in an epoch, the device is determined to have been worn for the entire epoch.

There are some limitations to the algorithm around its ability to detect that the device is worn when the user is very still. If the user stays sufficiently still, the worn detection can give incorrect results. This could be mitigated through the use of an additional sensor to detect contact with the skin or through additional analysis to detect the events associated with the WWU being taken off or put on.

### 5.2 Activity levels

**Figure 5:**
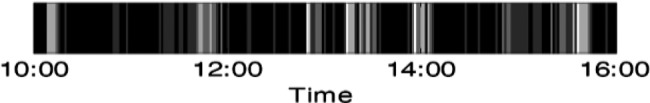
Activity bar showing activity levels where black is zero activity and white is level 5

Fig. 5 shows an activity bar for the time the researcher wore the WWU. There are clearly periods of greater intensity activity, around 11:30–12:00, for example, and periods of lower intensity activity, 14:00–15:00, for example.

**Figure 6:**
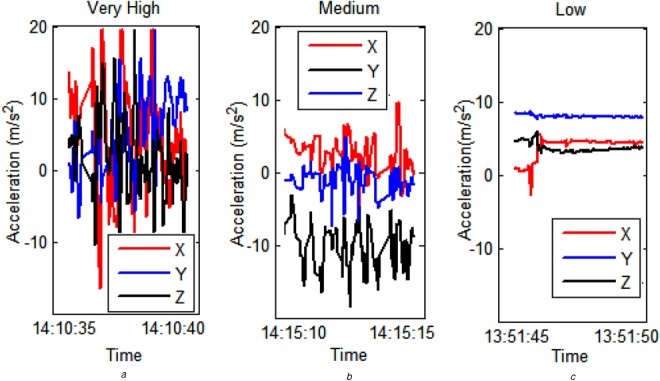
Activity traces from WWU showing activity level classifications derived by our system *a* Very high *b* Medium *c* Low activity

The periods of activity in this plot match well to the known activity of the participant over the day. Coffee breaks at 11:30 and 15:30 result in higher levels of activity being registered. Lunch at around 13:00 results in two periods of high intensity activity with a low intensity period in the middle. This is consistent with walking, sitting and eating, and walking again. Fig. 6 shows examples of accelerometry recorded from the WWU and classified by the activity level detection algorithm. Acceleration is shown in three axes prior to the removal of the gravity component. A visual analysis of the graphs in Fig. 5 shows that there are varying degrees of activity across the graphs and is the expected result given that we are classifying from RMS values. Fig. 6*a* shows very high activity, Fig. 6*b* medium activity and Fig. 6*c* low activity.

High intensity periods correlate to greater amounts of movement, such as walking, whereas lower intensity periods correlate to more sedate activities, such as desk work. Very short duration, high intensity activity in a minute of otherwise low intensity activity tends to result in a medium activity score. A good example of this is retrieving a document from the printer where the participant is seated, gets up and walks to the printer and then returns to their desk.

The results demonstrate that accelerometer data can be processed to extract measures of activity level and that, these can be categorised easily for display purposes. Furthermore, these category values map sensibly onto different intensities of activity.

As we are interested in identifying the step counts only during the active periods and not when the person is less active, identifying the step counts when activity level greater than or equal to 3 works for our purpose. A limitation of using this approach is that there could be a few other activities (e.g. washing up) with a lot of wrist-movement, which could provide an activity level greater than or equal to 3.

### 5.3 Step counts

The mean percentage errors calculated for each data set are given in Table 1. The TOST showed that the WWU data gave step counts equivalent to the truth for normal walking and walking at 1 Hz. However, the step counts for walking up and down stairs were not equivalent to the truth, although setting the equivalence interval as 5% either side of the truth gives equivalence.

**Table 1:** Mean percentage error for the different datasets

Dataset type and sampling rate	Mean percentage error, %
normal walking (50 Hz)	1.25
slow walking (50 Hz)	0.60
walking down stairs (50 Hz)	5.03
walking up stairs (50 Hz)	3.38
normal walking (16 Hz)	1.50
normal walking (5 Hz)	2.85

As can be seen in Table 1, the percentage errors for step counts of data taken at 50 and 16 Hz are similar, but data taken at 5 Hz is larger, 2.85%. This is supported by the TOST that was performed, which said that the errors for step counts at 16 Hz were equivalent to 0 (and also equivalent to those taken at 50 Hz) whereas the errors for step counts at 5 Hz were not equivalent to 0 (or to those taken at 50 Hz). This suggests that the WWU accelerometer sampling rate could be set to 16 Hz, for efficient processing.

The statistical analysis shows that the algorithm is accurate enough to detect steps from normal walking, and slow paced walking, to integrate into the WWU to be used as a pedometer. However, it is less accurate with walking up and down stairs so further work could be done on determining when this type of walking is occurring and counting steps accordingly.

### 5.3 Limitations

It is recognised that there are some limitations to the work presented in this Letter. The algorithms presented here have been tested with normal controls and with the small number of participants, as is standard practice for testing at this stage. Subsequent work will be performed to evaluate the algorithms and device with a larger number of target users.

## 6 Conclusion

This Letter presents activity parameters (device worn status, activity levels and step counts) extracted from the WWU and the algorithms that are used for this. The statistical analysis shows that the algorithm is accurate enough to detect steps from normal walking, and slow paced walking, to integrate into the WWU to be used as a pedometer.

It should be noted, however, that the use of the WWU is dependent on the user wearing the device and acting in concordance with the usage requirements. This could present potential issues if the device is not worn and data are lost. This is minimised in the WWU, and USEFIL, in two ways. Firstly, the USEFIL system is voluntary and thus expected to be used by users who are motivated to act in concordance with the requirements. Secondly, the watch form factor is familiar, which is expected to reduce the barrier to usage.

Further work could be performed to develop an estimate of calorific energy expenditure by drawing both on the raw accelerometer data and step count data, as well as user specific parameters such as height and weight. Such an estimate would require validation against a gold standard calorimeter and could provide a better picture of activity than activity level and step count alone.

The results are a promising step in the development of the WWU for the USEFIL system and show that a number of algorithms can be packaged into a smart-watch to obtain an indication of a person's PA. It is envisaged that this will be developed further with a view towards detecting and classifying ADL. The steps, along with other parameters such as energy expenditure, activity level, postural transition and activity classification, could provide information about the onset of a health condition or deterioration of health of an older person at an early stage to provide support. This work could also be extended to monitor the ability of a person to perform their ADL and also identify incidents such as falls. This can be used to assist in the care giving for such a person.
